# Treatment options for dogs diagnosed with sinonasal aspergillosis

**DOI:** 10.18849/ve.v8i4.615

**Published:** 2023-10-11

**Authors:** Hannah Belton+, Jessica Lobb+

**Keywords:** SINONASAL ASPERGILLOSIS, SNA, DEBRIDEMENT, CANINE, TREATMENT, DOGS

## Abstract

**PICO question:**

In dogs diagnosed with sinonasal aspergillosis (SNA) does inclusion of debridement in the treatment protocol improve clinical outcomes compared to dogs treated without debridement?

**Clinical bottom line:**

**Category of research:**

Treatment.

**Number and type of study designs reviewed:**

No papers that directly addressed the PICO were reviewed. Four retrospective case series which partially addressed the PICO question were discussed in the appraisal.

**Strength of evidence:**

Zero.

**Outcomes reported:**

None.

**Conclusion:**

No conclusions can be made based on the current level of evidence, however the studies discussed in the appraisal suggest that there may be an association between the inclusion of debridement in the treatment protocol for SNA and an improved clinical outcome. Undertaking further higher-level studies (comparative, blind, prospective, randomised) would be required to confirm this. Ethical implications would not preclude such studies, for there is evidence that treatment protocols including topical deposition of antifungal agents alone are efficacious.

## How to apply this evidence in practice

The application of evidence into practice should take into account multiple factors, not limited to: individual clinical expertise, patient’s circumstances and owners’ values, country, location or clinic where you work, the individual case in front of you, the availability of therapies and resources.

Knowledge Summaries are a resource to help reinforce or inform decision making. They do not override the responsibility or judgement of the practitioner to do what is best for the animal in their care.

## Clinical scenario

A 6-year-old Golden Retriever has been diagnosed with Sinonasal Aspergillosis (SNA) following a history of chronic profuse bilateral mucopurulent nasal discharge and sneezing. You present two treatment options to the owner, a treatment protocol including debridement (surgical or minimally invasive techniques) and one without. The owner asks which treatment option will most likely result in a rapid and complete recovery and have a reduced chance of recurrence of the disease, in addition to questioning the necessity for invasive treatment protocols. You want to be able to advise the most efficacious treatment option for the owner.

## The evidence

There is currently no literature available that directly addresses the clinical question.

## Appraisal, application and reflection

The debridement of fungal plaques prior to the application of topical anti-fungal treatment has been proposed to improve the clinical outcome for dogs with sinonasal aspergillosis (SNA). Treatment protocols are varied, without one single efficacious treatment having been recognised, where in addition to the method of debridement being very variable, there is also debate over which antifungal has the greatest efficacy in the treatment of the disease, although it is beyond the scope of this Knowledge Summary to discuss this. Studies such as that by Billen et al. (2010), which evaluated the efficacy of the use of bifonazole as an adjunctive therapy to enilconazole infusion or as a sole therapy, have investigated this. Other studies have evaluated the efficacy of different topical therapy applications. The application of topical therapy has been proposed via several techniques including instillation of enilconazole via surgically implanted catheters directly into the nasal cavity and combined trephination, short clotrimazole soak, and application of clotrimazole cream to the frontal sinuses (Vedrine & Fribourg-Blanc, 2018). The purpose of this Knowledge Summary was to look for evidence regarding whether including debridement in the treatment protocol, whether that be surgical debridement or a more minimally invasive technique, provides a better clinical outcome compared to protocols with medical treatment alone.

A thorough literature search concluded that there were no publications reporting a direct comparison between protocols including debridement compared to those without. Four recent retrospective papers (Balber et al., 2018; Hazuchova et al., 2017; Vangrinsven et al., 2018; and Vedrine & Fribourg-Blanc, 2017) partially address the PICO question and include debridement within the treatment protocol but provide weak evidence with inherent limitations. Small study sizes in case series investigations do not allow for power analysis. In addition, the study design of retrospective case series results in many weaknesses. Causation cannot be determined from the results of these studies, only association, and the study design is prone to selection bias and providing a non-representative population. Control of confounding variables is lacking in the patients, for example, in the study by Vangrinsven et al. (2018) where evidence of cribriform plate lysis was only assessed in 15/48 (31%) of cases. Cribriform plate lysis is thought to result in poorer treatment outcomes (Belda et al., 2018).

Assessment of the impact of complete debridement in the study conducted by Vangrinsven et al. (2018) concluded that first treatment failure was lower in dogs with complete debridement 12/48 (25%) compared to dogs with partial debridement 28/48 (60%) (OR 0.24, CI 0.07–0.85, P = 0.03). Treatment success was reported 46/48 dogs (96%), with no dog receiving more than two infusion protocols, supporting the inclusion of debridement in treatment protocols for SNA. Eighty-three dogs diagnosed with SNA from October 2006 until February 2015 based on compatible clinical signs and endoscopic identification of fungal plaques with turbinate destruction were included in the study. Vangrinsven et al. (2018) compared the visual resolution of fungal plaque lesions between a 1-hour enilconazole and bifonazole infusion protocol (an original protocol implemented before December 2011) to a simplified 15-minute enilconazole infusion protocol (protocol implemented since December 2011) followed with oral itraconazole. Debridement using per-endoscopic forceps was then performed before infusion in both groups. Assessment of completeness of debridement and the impact on first treatment success was determined by the presence or absence of macroscopic fungal plaques at the end of the procedure. They also proposed that where debridement was considered complete but there was subsequent first treatment failure, this may have been due to residual disease not identified by visual assessment rather than failure of the treatment protocol. A change in assessment methods of debridement completeness could counteract this problem.

Studies by Ballber et al. (2018) and Vedrine & Fribourg-Blanc (2018) evaluated the clinical outcomes of similar treatment protocols. Inclusion criteria differed between each study. Ballber et al. (2018) required destructive rhinitis on computed tomography with at least one of the following: positive fungal culture, characteristic fungi on histopathology or identification of fungal plaques rhinoscopically. Vedrine & Fribourg-Blanc (2018) required the presence of clinical signs and macroscopic fungal plaques on endoscopy associated with turbinate destruction and / or fungal culture.

The treatment protocol for both included the use of a minimally invasive debridement technique combined with topical application of 1% clotrimazole cream. The 12 patients included in the study by Ballber et al. (2018) underwent endoscopic debridement using biopsy forceps followed by irrigation through a catheter passed over a guide wire. This treatment protocol was concluded by instilling 1% clotrimazole cream into the frontal sinus and nasal cavity via the catheter. This was repeated at 2-week intervals until there was no gross evidence of disease. A similar protocol was used in the 10 patients in the Vedrine & Fribourg-Blanc (2018) study. Endoscopically guided fungal plaque debridement was performed, using both forceps and suction catheters. 1% clotrimazole cream was instilled via an oxygen catheter without prior irrigation.

First treatment success was achieved in 2/12 (16.7%) patients in the study by Ballber et al. (2018), in comparison to a reported outcome of 5/10 (50%) patients in the study by Vedrine & Fribourg-Blanc (2018). However, differences in definition of disease resolution could account for the disparity noted here, concluding that the results are not directly comparable. Ballber et al. (2018) required additional negative fungal culture and lack of identifiable fungal elements on histopathology to confirm disease resolution compared to the single criteria of absence of visual fungal plaques used by Vedrine & Fribourg-Blanc (2018). The clinical significance of the association between positive culture and subsequent return of clinical signs cannot, however, be determined as both studies reported similar disease recurrence rates with 2/12 (16.7%) and 2/10 (20%) dogs respectively.

A final retrospective case series (Hazuchova et al., 2017) included two groups of patients, one group (n = 42) of which had undergone surgical trephination to perform debridement and one group (n = 22) in which the dogs underwent per-endoscopic debridement using forceps. In both groups this was followed by flushing and suction until visible fungal material and necrotic tissue was removed and the instillation of 1% clotrimazole cream. This study did not find evidence to suggest that the use of surgical trephination resulted in a change in the number of treatments required for resolution of disease among dogs with successful outcomes compared to endoscopic debridement. Resolution was achieved in 58/64 dogs (90.6%), defined by the absence of visible fungal plaque with no or negligible amounts of necrotic turbinate material present on recheck rhinoscopy (38/58) or based on telephone interview (20/58). A single treatment was required for clinical resolution in 42/58 dogs (72.4%). Treatment complication rate was 12/64 (18.8%) with deaths possibly attributable to SNA or its treatment were included. Reinfection was diagnosed in seven dogs with a median of 18 months (range 12–30 months) post-treatment. Successful treatment was completed again in all seven of these dogs.

For further assessment of the inclusion of debridement in the treatment protocol for SNA in dogs, further studies which are prospective, blind, and randomised must be performed which are higher on the hierarchy of evidence than is currently available. It seems appropriate that control studies could be performed ethically and without welfare concerns, where there is evidence to suggest that alternative treatments without the inclusion of debridement do offer good outcomes. Currently, there is insufficient evidence to make any meaningful clinical conclusions on the usefulness of the technique, although case reports provide the evidence to prompt further study on the topic.

## Methodology

**Search strategy:** 

Databases searched and dates covered:	CAB Abstracts on OVID Platform 1973 to Week 14 2023 Medline on OVID Platform 1946–present Web of Science 1900–present
Search strategy:	CAB Abstracts: exp dogs/ (dog* or canine*).mp. [mp=abstract, title, original title, broad terms, heading words, identifiers, cabicodes] 1 or 2 Aspergillosis/ or aspergillus/ (Aspergillosis or Aspergilloses or aspergillus).mp. [mp=abstract, title, original title, broad terms, heading words, identifiers, cabicodes] 4 or 5 Debride*.mp. [mp=abstract, title, original title, broad terms, heading words, identifiers, cabicodes] 3 and 6 and 7 Medline: Dogs/ (dog* or canine*).mp. [mp=title, abstract, original title, name of substance word, subject heading word, floating sub-heading word, keyword heading word, organism supplementary concept word, protocol supplementary concept word, rare disease supplementary concept word, unique identifier, synonyms] 1 or 2 Aspergillosis/ (Aspergillosis or Aspergilloses or aspergillus).mp. [mp=title, abstract, original title, name of substance word, subject heading word, floating sub-heading word, keyword heading word, organism supplementary concept word, protocol supplementary concept word, rare disease supplementary concept word, unique identifier, synonyms] 4 or 5 Debride*.mp. 3 and 6 and 7 **Web of Science: **(dog* or canine*) (All Fields) and (aspergillosis OR aspergillosis OR aspergillus OR aspergillus fumigatus) (All Fields) and debride* (All Fields)
Dates searches performed:	14 Apr 2023

**Exclusion / inclusion criteria:** 

Exclusion:	Not written in English. Debridement not used in treatment protocol.
Inclusion:	Articles available in English that were relevant to the PICO.

**Search outcome:** 

Database	Number of results	Excluded – Does not answer PICO	Excluded – Duplicates	Excluded – Not written in English	Total relevant papers
CAB Abstracts	10	9	0	1	0
Medline	10	2	8	0	0
Web of Science	21	12	9	0	0
Total relevant papers when duplicates removed					0

### Acknowledgements

The authors acknowledge the contribution of Victoria Black and Emma Place in the preparation of this manuscript.

### ORCiD

Hannah Belton: https://orcid.org/0000-0002-1525-1043 Jessica Lobb: https://orcid.org/0000-0003-4737-1346

### Conflict of interest

The authors declare no conflicts of interest.

## Disclaimer

Knowledge Summaries are a peer-reviewed article type which aims to answer a clinical question based on the best available current evidence. It does not override the responsibility of the practitioner. Informed decisions should be made by considering such factors as individual clinical expertise and judgement along with patient’s circumstances and owners’ values. Knowledge Summaries are a resource to help inform and any opinions expressed within the Knowledge Summaries are the author’s own and do not necessarily reflect the view of the RCVS Knowledge. Authors are responsible for the accuracy of the content. While the Editor and Publisher believe that all content herein are in accord with current recommendations and practice at the time of publication, they accept no legal responsibility for any errors or omissions, and make no warranty, express or implied, with respect to material contained within. For further information please refer to our Terms of Use.
